# Genetic diversity and population structure analysis of Kala bhat (*Glycine max* (L.) Merrill) genotypes using SSR markers

**DOI:** 10.1186/s41065-017-0030-8

**Published:** 2017-04-27

**Authors:** Yegappa Hipparagi, Rakesh Singh, Debjani Roy Choudhury, Veena Gupta

**Affiliations:** 1 Division of Plant Genetic Resources, ICAR-Indian Agricultural Research Institute, New Delhi, 110 012 India; 2 Division of Genomic Resources, ICAR-National Bureau of Plant Genetic Resources, New Delhi, 110 012 India; 3 Division of Germplasm Conservation, ICAR-National Bureau of Plant Genetic Resources, New Delhi, 110 012 India

**Keywords:** Soybean, Genetic diversity, SSR markers, Seed colour

## Abstract

**Background:**

Kala bhat (Black soybean) is an important legume crop in Uttarakhand state, India, due to its nutritional and medicinal properties. In the current study, the genetic variabilities present in Kala bhat were estimated using SSR markers and its variability was compared with other improved soybean varieties cultivated in Uttarakhand state, India.

**Results:**

Seventy-five genotypes cultivated in different districts of Uttarakhand were collected, and molecular analysis was done using 21 SSR markers. A total of 60 alleles were amplified with an average of 2.85 alleles per locus. The mean value of gene diversity and PIC was estimated to be 0.43 and 0.36, respectively. The unrooted phylogenetic tree grouped soybean genotypes into three major clusters, where, yellow seed coat (improved varieties) genotypes were grouped in one cluster, while reddish brown (improved varieties) and Kala bhat showed intermixing. Population structure divided the soybean genotypes into six different populations. AMOVA analysis showed 12% variance among the population, 66% variance among individual and 22% variance was observed within individuals. Principal Coordinate Analysis (PCoA) also showed that yellow seed coat genotypes were grouped in one cluster, whereas, the Kala bhat showed scattered distribution and few genotypes of Kala bhat showed grouping with red and yellow genotypes.

**Conclusions:**

The different genetic diversity parameters used in the present study indicate that Kala bhat genotypes were more diverse than the yellow seed coat and brown seed coat colour genotypes. Therefore, Kala bhat genotypes can be a good source for the soybean breeding programme due to its better genetic diversity as well as its medicinal properties.

## Background

Soybean (Glycine max (L.) Merr) is an important legume crop which contains 37–42% protein, 17–24% oil and 35% carbohydrates [1], that served as an excellent source of oil and protein for human consumption and animal feed. The wild and cultivated soybeans showed significant phenotypic diversity but the small reproductive difference, and they have very similar genomes in both its size and content [2]. Soybean is grown under varied climatic conditions and geographical locations in India. It occupies an area of 10.8 million hectare and accounting to a production of 11.5 million tone with the productivity of 1065 kg/ha [3]. A potential source of protein and oil makes soybeans a large share in human nutrition, and also improves soil fertility therefore; soybean is also an important crop for research [4].

In soybean, evaluation of genetic diversity is enhanced by the use of DNA markers. Researchers have studied the genetic divergence among soybean genotypes for various agronomic traits [5–8] with molecular markers [9–11]. Among different DNA markers, restriction fragment length polymorphisms (RFLPs), random amplified polymorphic DNAs (RAPDs), amplified fragment length polymorphisms (AFLPs), single nucleotide polymorphisms (SNPs) and microsatellites or simple sequence repeats (SSRs) have been extensively used in soybean, each with its own advantages and limitations [12–17].

Black seed coat soybean, locally known by different names such as Bhat, Bhatmash and Kala bhat is grown in Kumaon and Garhwal region and in frontiers of Uttarakhand state [18]. In Uttarakhand, these soybean varieties are commonly known as Kala Bhat. It is believed that soybean was introduced by traders via Myanmar from Indonesia. As a result, it has been traditionally grown on a small scale in states like Himachal Pradesh, Kumaon and Garhwal hills of Uttarakhand, East Bengal, Khasi hills and small parts of central India. Kala bhat is also considered as the treasure trove of different medicinal properties. Kala bhat and its products are the richest sources of iso-flavones. Kala bhat, in Uttarakhand is grown in 5734 ha area, with a production and productivity is 5636 tonne and 9.82 q/ha, respectively (Anonymous, 2011). A traditional cultivar of Kala bhat is much low yielder than normal soybean varieties hence this can be improved further by crossing with diverse exotic as well as indigenous germplasm. Morphological characterization of 21 soybean cultivars was done by Oda et al. [19] and 24 Kala bhat genotypes was done by Bhartiya et al. [20].

Analyses of the genetic variation and population structure of Kala bhat genotypes are important for their effective conservation and utilization of the valuable genetic resource. The present study was done to estimate the genetic variability and population structure present in Kala bhat cultivated in Uttarakhand state using SSR markers, as the information on the level of diversity present in local landraces (Kala bhat) and population structure had not been studied systematically. The genetic diversity of Kala bhat was also compared with other improved soybean varieties cultivated in Uttarakhand.

## Methods

### Collection of plant materials

Seeds of 75 soybean genotypes were procured from NBPGR regional station located at Bhowali, Uttarakhand, India. The Seeds were sown in pots under controlled conditions inside the Green house of NBPGR, New Delhi. Black seed coat genotypes were the landraces (Kala bhat) whereas, reddish-brown and yellowish-white genotypes were improved varieties, which were introduced earlier and naturalized as the population in that agro-ecological region. The leaf samples were collected at 3–4 leaves stage for DNA isolation. The details of each genotype along with passport data, National ID, i.e. Indigenous Collection (IC) number, cultivar name, seed colour, district, region and state are given in Table 1.

**Table 1:** List of Soybean genotypes used in the study with their cultivar name, IC numbers, seed coat colour, district, region and state

S. No.	Cultivar name	IC numbers	Seed coat colour	District	Region	State
1	Bhatt	IC281596	Imperfect black	Bageshwar	Kumaon	Uttarakhand
2	Soybean	IC281602	Yellowish white	Bageshwar	Kumaon	Uttarakhand
3	Bhatt	IC281616	Imperfect black	Chamoli	Garhwal	Uttarakhand
4	Soybean	IC281618	Yellowish white	Almora	Kumaon	Uttarakhand
5	Soybean	IC281629	Yellowish white	Almora	Kumaon	Uttarakhand
6	Soybean	IC281644	Yellowish white	Almora	Kumaon	Uttarakhand
7	Soybean	IC281652	Yellowish white	Almora	Kumaon	Uttarakhand
8	Soybean	IC281655	Yellowish white	Almora	Kumaon	Uttarakhand
9	Soybean	IC281671	Yellowish white	Almora	Kumaon	Uttarakhand
10	Soybean	IC281684	Yellowish white	Almora	Kumaon	Uttarakhand
11	Soybean	IC281694	Yellowish white	Tehri	Garhwal	Uttarakhand
12	Kala bhatt	IC281815	Imperfect black	Almora	Kumaon	Uttarakhand
13	Bhatt	IC281838	Imperfect black	Almora	Kumaon	Uttarakhand
14	Soybean	IC281843	Yellowish white	Almora	Kumaon	Uttarakhand
15	Soybean	IC316141	Yellowish white	Bhowali	Kumaon	Uttarakhand
16	Bhatt	IC316142	Imperfect black	Bhowali	Kumaon	Uttarakhand
17	Soybean	IC316154	Yellowish white	Bhowali	Kumaon	Uttarakhand
18	Bhatt	IC316155	Imperfect black	Nainital	Kumaon	Uttarakhand
19	Bhatt	IC316163	Imperfect black	Nainital	Kumaon	Uttarakhand
20	Kala soybean	IC316170	Imperfect black	Almora	Kumaon	Uttarakhand
21	Bhatt	IC316171	Imperfect black	Almora	Kumaon	Uttarakhand
22	Kala soybean	IC316172	Imperfect black	Almora	Kumaon	Uttarakhand
23	Bhatt	IC316178	Imperfect black	Almora	Kumaon	Uttarakhand
24	Soybean	IC316181	Yellowish white	Bhowali	Kumaon	Uttarakhand
25	Soybean	IC316182	Yellowish white	Nainital	Kumaon	Uttarakhand
26	Bhatt	IC316183	Imperfect black	Nainital	Kumaon	Uttarakhand
27	Bhatt	IC316184	Imperfect black	Nainital	Kumaon	Uttarakhand
28	Bhatt	IC316186	Imperfect black	Nainital	Kumaon	Uttarakhand
29	Soybean	IC316188	Yellowish white	Nainital	Kumaon	Uttarakhand
30	Kala bhatt	IC316192	Imperfect black	Nainital	Kumaon	Uttarakhand
31	Kala soybean	IC316193	Imperfect black	Almora	Kumaon	Uttarakhand
32	Bhatt	IC317428	Imperfect black	Chamoli	Garhwal	Uttarakhand
33	Bhatt	IC317431	Yellowish white	Chamoli	Garhwal	Uttarakhand
34	Bhatt	IC317437	Imperfect black	Chamoli	Garhwal	Uttarakhand
35	Bhatt	IC317465	Reddish brown	Chamoli	Garhwal	Uttarakhand
36	Soybean	IC317578	Yellowish white	Dehradun	Garhwal	Uttarakhand
37	Soybean	IC317581	Yellowish white	Dehradun	Garhwal	Uttarakhand
38	Bhatt	IC317660	Imperfect black	Dehradun	Garhwal	Uttarakhand
39	Bhatt	IC317663	Imperfect black	Dehradun	Garhwal	Uttarakhand
40	Soybean	IC337280	Yellowish white	Pauri	Garhwal	Uttarakhand
41	Bhatt	IC338509	Imperfect black	Almora	Kumaon	Uttarakhand
42	Bhatt	IC338622	Imperfect black	Pauri	Garhwal	Uttarakhand
43	Bhatt	IC338626	Imperfect black	Nainital	Kumaon	Uttarakhand
44	Soybean	IC338702	Imperfect black	Champawat	Kumaon	Uttarakhand
45	Soybean	IC338713	Imperfect black	Champawat	Kumaon	Uttarakhand
46	Soybean	IC338717	Imperfect black	Champawat	Kumaon	Uttarakhand
47	Soybean	IC338720	Yellowish white	Champawat	Kumaon	Uttarakhand
48	Soybean	IC338729	Imperfect black	Champawat	Kumaon	Uttarakhand
49	Soybean	IC338732	Reddish brown	Champawat	Kumaon	Uttarakhand
50	Soybean	IC338749	Imperfect black	Champawat	Kumaon	Uttarakhand
51	Soybean	IC419875	Yellowish white	Chamoli	Garhwal	Uttarakhand
52	Bhatt	IC419896	Imperfect black	Chamoli	Garhwal	Uttarakhand
53	Bhatt	IC419909	Imperfect black	Chamoli	Garhwal	Uttarakhand
54	Kala bhatt	IC430009	Imperfect black	Bageshwar	Kumaon	Uttarakhand
55	Soybean	IC430038	Yellowish white	Bageshwar	Kumaon	Uttarakhand
56	Soybean	IC430041	Yellowish white	Bageshwar	Kumaon	Uttarakhand
57	Soybean	IC430063	Yellowish white	Bageshwar	Kumaon	Uttarakhand
58	Kala bhatt	IC430066	Imperfect black	Bageshwar	Kumaon	Uttarakhand
59	Soybean	IC430075	Imperfect black	Bageshwar	Kumaon	Uttarakhand
60	Soybean	IC430076	Yellowish white	Bageshwar	Kumaon	Uttarakhand
61	Black bhatt	IC436967	Imperfect black	Rudraprayag	Garhwal	Uttarakhand
62	Bhatt	IC444239	Imperfect black	Pithoragarh	Kumaon	Uttarakhand
63	Bhatt	IC444241	Imperfect black	Pithoragarh	Kumaon	Uttarakhand
64	Bhatt	IC444249	Reddish brown	Pithoragarh	Kumaon	Uttarakhand
65	Kala bhatt	IC469759	Imperfect black	Champawat	Kumaon	Uttarakhand
66	Kala bhatt	IC469767	Imperfect black	Champawat	Kumaon	Uttarakhand
67	Kala bhatt	IC469833	Imperfect black	Champawat	Kumaon	Uttarakhand
68	Soybean	IC469881	Yellowish white	Pithoragarh	Kumaon	Uttarakhand
69	Kala bhatt	IC469902	Imperfect black	Pithoragarh	Kumaon	Uttarakhand
70	Kala bhatt	IC524256	Imperfect black	Pauri	Garhwal	Uttarakhand
71	Bhatt	IC538013	Imperfect black	Nainital	Kumaon	Uttarakhand
72	Bhatt	IC538042	Imperfect black	Nainital	Kumaon	Uttarakhand
73	Bhatt	IC538070	Yellowish white	Champawat	Kumaon	Uttarakhand
74	Kala bhatt	IC548612	Imperfect black	Almora	Kumaon	Uttarakhand
75	Kala bhatt	IC548623	Imperfect black	Chamoli	Garhwal	Uttarakhand

### DNA extraction

Five grams of young fresh leaves were crushed in liquid nitrogen using a motor pestle and DNA was isolated using CTAB method [21]. The DNA quality was first checked on 0.8% agarose gel and then quantified using Nanodrop (Thermo Fisher, USA). A working concentration of 10 ng/μl DNA stock was prepared for all the 75 soybean genotypes and stored at 4 °C.

### Genotyping of soybean genotypes using SSR markers

Total 51 SSR markers were selected for initial screening. Gradient PCR was done for each primer with selected soybean samples to standardize the temperature for amplification (Ta). 21 SSR primers (Table 2) out of 51 showed good amplification and were considered for further study. These 21 primers were subjected to PCR analysis with 75 soybean samples.

**Table 2:** List of SSR primers used for genotyping of 75 soyabean genotypes along with their product size, no. of alleles amplified, gene diversity, heterozygosity and PIC value

Marker	Size (bp)	Forward primer	Reverse primer	Major allele frequency	Allele No	Gene diversity	Heterozygosity	PIC
sat005	141	TATCCTAGAGAAGAACTAAAAAA	GTCGATTAGGCTTGAAATA	0.6000	2.0000	0.4800	0.0571	0.3648
sat385	310	AATCGAGGATTCACTTGAT	CATTGGGCCACACAACAAC	0.6081	2.0000	0.4766	0.0000	0.3630
sat415	297	GCGTCTCCCTTAATCTTCAAGC	GCGTGTGACGGTTCAAAATGATAGTT	0.6197	3.0000	0.4999	0.0000	0.4104
sat577	119	CAAGCTTAAGTCTTGGTCTTCTCT	GGCCTGACCCAAAACTAAGGGAAGTG	0.6884	3.0000	0.4637	0.0145	0.4039
sat180	242	TCGCGTTTGTCAGC	TTGATTGAAACCCAACTA	0.3542	4.0000	0.7210	0.1250	0.6689
sat277	243	GGTGGTGGCGGGTTACTATTACT	CCACGCTTCAGTTGATTCTTACA	0.6923	2.0000	0.4260	0.0000	0.3353
sat422	250	ATTAGGGGAGGGGAGGTAAAAAGT	TGAAGGCCCGATATCCAAATAAA	0.5208	3.0000	0.5651	0.0139	0.4742
sat600	195	GCGCAGGAAAAAAAAACGCTTTTATT	GCGCAATCCACTAGGTGTTAAT	0.5625	4.0000	0.6189	0.1389	0.5753
sat389	232	GCGGCTGGTGTATGGTGAAATCA	GCGCCAAAACCAAAAGTTATATC	0.9400	2.0000	0.1128	0.0400	0.1064
sat411	97	TGGCCATGTCAAACCATAACAACA	GCGTTGAAGCCGCCTACAAATATAAT	0.5462	2.0000	0.4957	0.0154	0.3729
sat554	261	GCGATATGCTTTGTAAGAAAATTA	GCGCAAGCCCAAATATTACAAATT	0.5530	4.0000	0.5874	0.0758	0.5200
sat285	236	GCGACATATTGCATTAAAAACATACTT	GCGGACTAATTCTATTTTACACCAACAAC	0.9400	2.0000	0.1128	0.0133	0.1064
sat183	240	TAGGTCCCAGAATTTCATTG	CACCAACCAGCACAAAA	0.6800	2.0000	0.4352	0.0000	0.3405
sat431	250	GCGTGGCACCCTTGATAAATAA	GCGCACGAAAGTTTTTCTGTAACA	0.4857	3.0000	0.6171	0.0571	0.5409
sat247	221	GCGCCCATGTGGCTATTTCTTTATTT	GCGGATCAATAATAAACAAAGTGACAA	0.8933	2.0000	0.1906	0.0000	0.1724
sat175	163	GACCTCGCTCTCTGTTTCTCAT	GGTGACCACCCCTATTCCTTAT	0.8733	2.0000	0.2212	0.0133	0.1968
sat306	212	GCGCTTAAGGACACGGATGTAAC	GCGTCTCTTTCGATTGTTCTATTAG	0.5074	2.0000	0.4999	0.9853	0.3749
sat255	141	GCGCTTTTAGCGTCGTCTGGC	TACCCCTCTCTTATTCTTCTT	0.8493	3.0000	0.2597	0.0274	0.2324
sat584	189	GCGCCCAAACCTATTAAGGTATGAACA	GCGGGTCAGAAGATGCTACCAAACTCT	0.7719	2.0000	0.3521	0.0000	0.2901
sat420	232	GCGTATTCAGCAAAAAAATATCAA	TTATCGCACGTGTAAGGAGACAAAT	0.7800	2.0000	0.3432	0.0133	0.2843
sat478	190	CAGCCAAGCAAAAGATAAATAATA	TCCCCCACAAGAGAACAAGAAGGT	0.5423	4.0000	0.6341	0.8592	0.5886
			Mean	0.6671	2.6190	0.4340	0.1166	0.3677

PCR reaction was set in a total volume of 10 μl containing 2 μl genomic DNA (10 ng/μl), 1 μl of 10X buffer, 0.8 μl of 25 mM MgCl_2_, 0.2 μl of 10 mM dNTPs, 0.2 μl of each primer (10 nmol), 0.2 μl of Taq DNA polymerase (Fermentas, Life Sciences, USA) and 5.6 μl distilled water. Amplification was performed in a thermocycler (G Storm, UK) using following program; Initial denaturation at 94 °C for 4 min followed by 36 cycles of 94 °C for 30 s, Ta for 45 s, 72 °C for 1 min and a final extension at 72 °C for 10 min. The amplified products were analyzed on 4% metaphor agarose gel for 4 h at a constant supply of 120 V. Gel pictures were recorded using gel documentation System (Alpha Imager®, USA).

### Statistical analysis

SSR bands generated near expected product size were scored visually for all 75 genotypes of Soybean. The band size of amplified products was determined by comparing with 100 bp DNA ladder (Fermentas, Life Sciences, USA). The SSR bands scored in soybean genotypes was subjected to statistical analysis. Major allele frequency, gene diversity, heterozygosity and polymorphic information content (PIC) for each locus for SSR markers were calculated using Power Marker 3.25 [22]. In addition, genetic distances across the soybean genotypes were calculated using Power Marker 3.25, and a phylogenetic tree was constructed and viewed in Mega version 6 [23] . Principle Coordinate Analysis (PCoA) and Analysis of Molecular Variance (AMOVA) were performed using software GenAlEx V6.5 [24]. The model-based program, STRUCTURE 2.3.3 [25] was used to infer the population structure. For each K, three replications were run. Each run was implemented over a burn-in period of 100,000 steps with 100,000 Monte Carlo Markov Chain replicates. The membership of each genotype was run for a range of genetic clusters from the value of K = 1 to 20 by taking admixture model and correlated allele frequency into account. LnPD derived for each K was then plotted to find the plateau of the ΔK values [26]. The “Structure harvester” program was used (http: //taylor0. biology.ucla.edu) to determine the final population. Venn diagram analysis was performed to identify common accessions between cluster and population using software Venny 2.1 [27].

## Results

Total 21 SSR primers were used for genetic diversity study of 75 soybean genotypes. A total of 60 alleles were amplified with an average of 2.85 alleles per locus. The number of alleles amplified per SSR primer varied from 2 to 4 (Table 2) and maximum numbers of alleles were amplified with primer Sat180, Sat600, Sat554 and Sat478 (four alleles). Gene diversity varied from 0.72 (Satt 180) to 0.11 (Satt 389 and Satt 285) with a mean value of 0.43. The heterozygosity ranged from 0.00 (Satt385, Satt415, Satt277, Satt183, Satt247, Satt584) to 0.98 (Satt 306). Major allele frequency was lowest for Satt180 (0.35) and maximum for Satt389 and Satt285 (0.94). The maximum PIC was observed for primer Satt 180 (0.66) and the minimum was observed for Satt285 and Satt389 (0.10) with a mean value of 0.36. (Table 2).

### Hierarchical cluster analysis

**Fig. 1:**
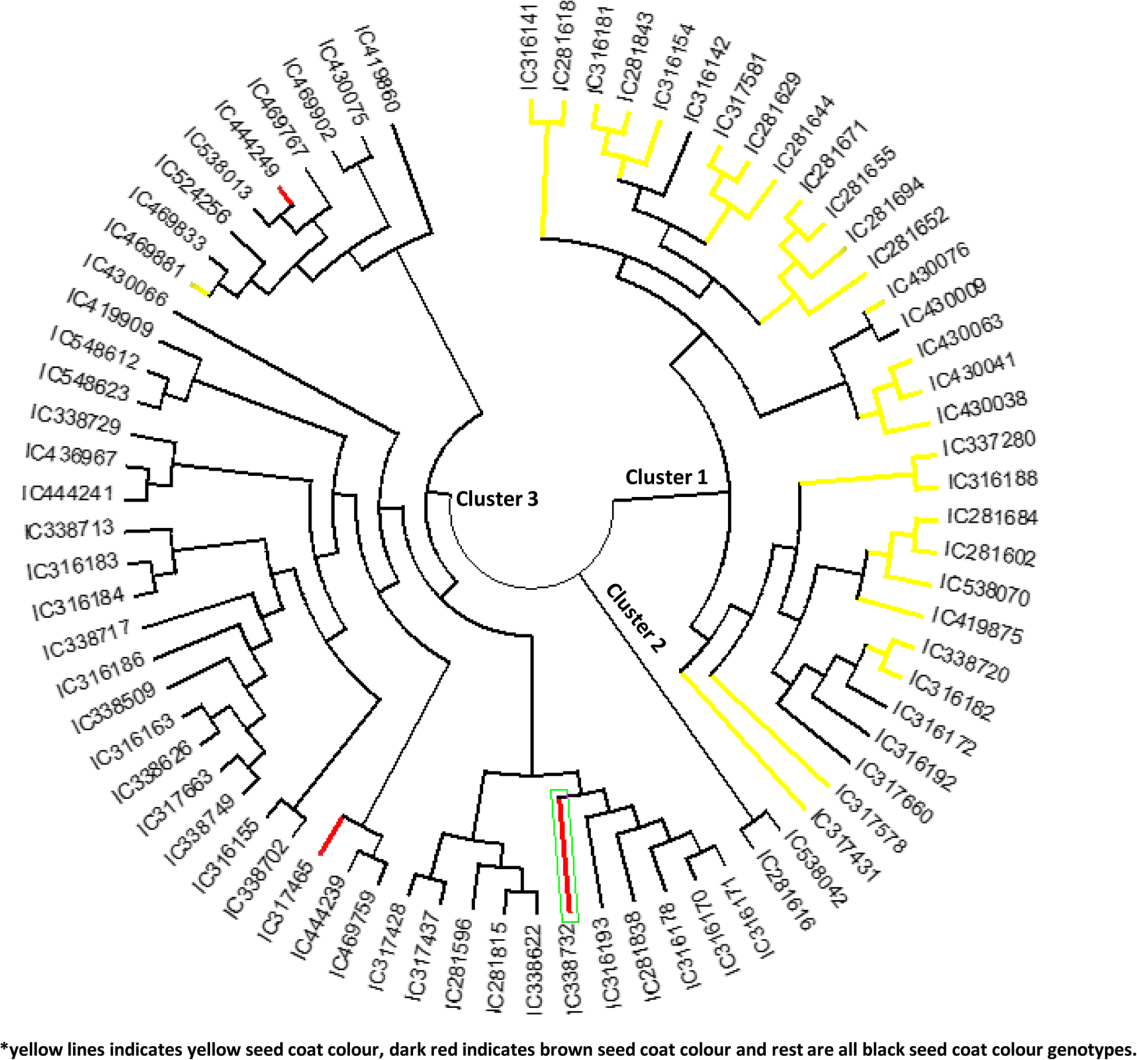
NJ tree of 75 soybean genotypes based on SSR markers

Soybean genotypes were grouped into three major clusters (Fig. 1). Kala bhat got distributed in all the three clusters whereas, brown seed coat colour soybean got grouped only into cluster3 that was mainly dominated by Kala bhat, which shows that there is mixing up of the genetic background between them. However yellow seeded soybeans were grouped into only cluster1 but five genotypes (IC316142, IC430009, IC316172, IC316192 and IC317660) of Kala bhat also grouped with yellow seed coat colour genotypes in cluster1. This hierarchical cluster analysis showed that Kala bhat is sharing genetic similarity with both, yellow and brown seed coat colour soybean, but, there is no sharing of genetic similarity between brown and yellow seed coat colour soybeans.

### Population structure

**Fig. 2:**
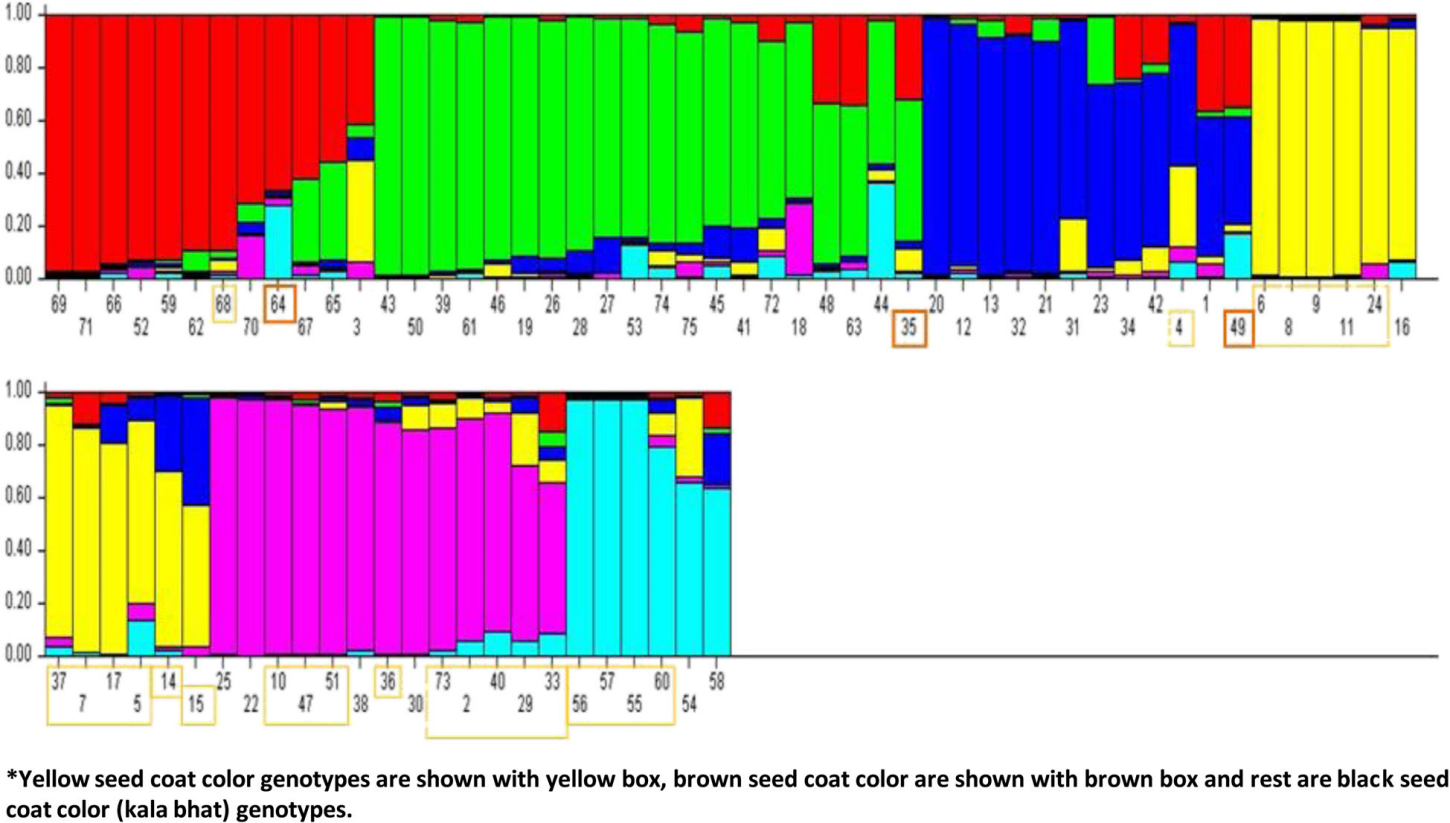
Population structure of 75 soybean genotypes based on SSR markers

**Fig. 3:**
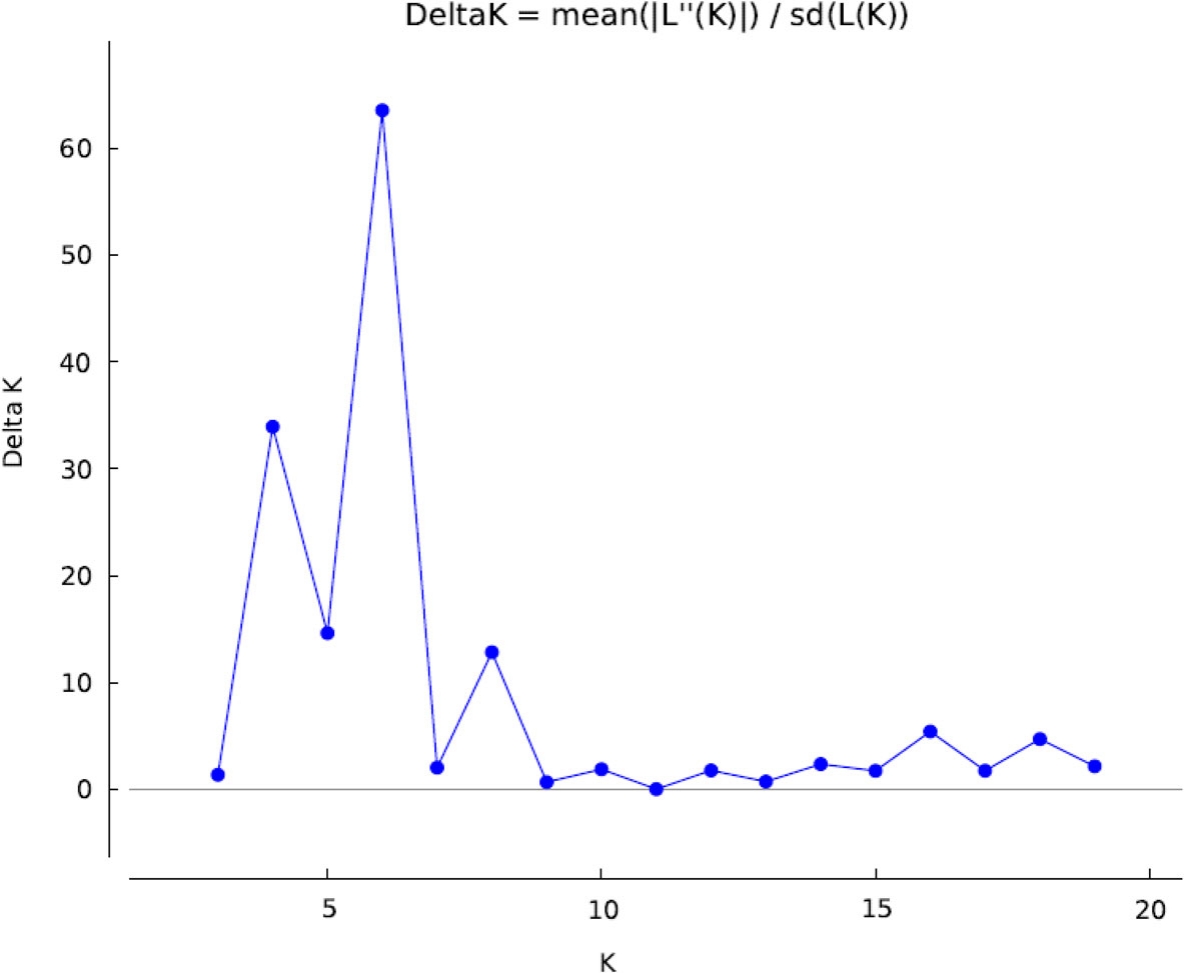
Estimation of population using LnP(D) derived Δk for k from 1 to20

The 75 soybean genotypes got distributed into six populations (Figs. 2 and 3). Seven pure and five admix individuals were present in population1; twelve pure and eight admix individuals were in population 2; five pure and seven admix individuals in population 3; eight pure and four admix individuals in population 4, 10 pure and three admix individuals in population 5, and three pure and three admix individuals in population 6. Mean Fst value for pop1, pop2, pop3, pop4, pop5 and pop6 were 0.464, 0.498, 0.332, 0.608, 0.345, and 0.688 respectively with a mean alpha value of 0.058. The allele frequency divergence among populations is given in Table 3. Average distances (expected heterozygosity) between individuals in the same cluster were between the range of 0.148 for cluster 6 and 0.378 for cluster 5. Population 1, 2 and 3 were dominated by Kala bhat and brown seed coat colour genotypes (highlighted with brown box) got distributed in all the three populations (Fig. 2) while, population 4, 5 and 6 were dominated by yellow seed coat colour genotypes (Fig. 2). Population structure based grouping supports the hierarchical cluster analysis and genotypes grouped in cluster1 corresponds to pop4,5 and 6 while genotypes grouped in cluster3 corresponds to pop1, 2 and 3.

**Table 3:** Allele-frequency divergence among populations computed using estimates of P (Model based approach)

	POP1	POP2	POP3	POP4	POP5	POP6
POP1	-	0.1944	0.1781	0.2448	0.1634	0.1877
POP2	0.1944	-	0.1609	0.2932	0.3036	0.2782
POP3	0.1781	0.1609	-	0.1892	0.2029	0.2013
POP4	0.2448	0.2932	0.1892	-	0.2197	0.1772
POP5	0.1634	0.3036	0.2029	0.2197	-	0.2044
POP6	0.1877	0.2782	0.2013	0.1772	0.2044	-

### Analysis of molecular variance (AMOVA)

Analysis of molecular variance (AMOVA) of soybean genotypes based on seed coat color was performed to analyze the distribution of genetic diversity between and within the populations. AMOVA analysis showed 12% diversity among populations, 22% diversity within individuals and a maximum of 66% diversity among individuals (Table 4).

**Table 4:** Summary of AMOVA for three soybean populations

Source	df	SS	MS	Est. Var.	%
Among Pops	10	164.219	16.422	0.628	12%
Among Indiv	66	528.158	8.002	3.443	66%
Within Indiv	74	86.000	1.162	1.162	22%
Total	150	778.377		5.188	100%

### Principal coordinate analyses (PCoA)

**Fig. 4:**
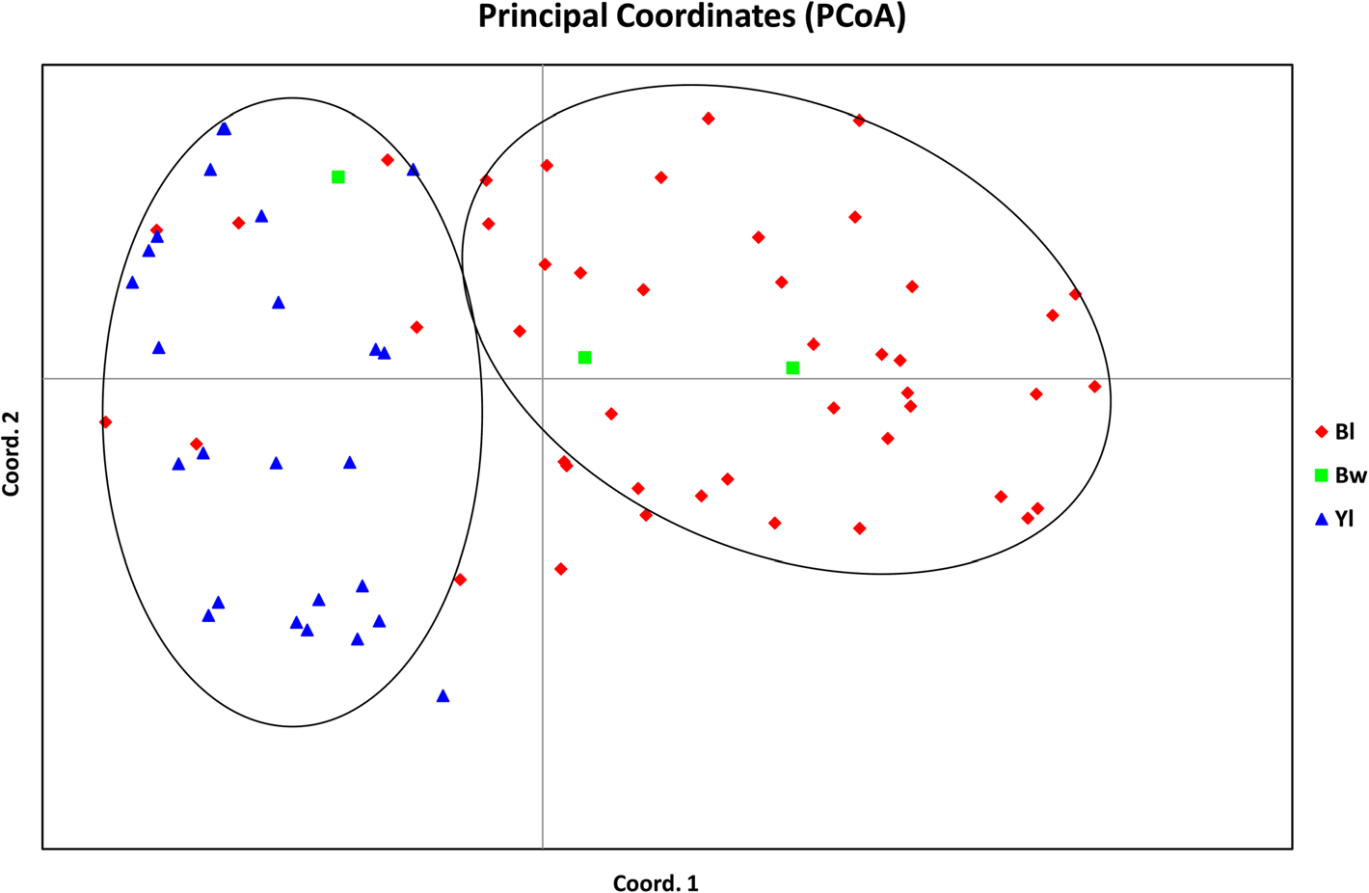
Principal Coordinate Analysis (PCoA) of 75 soybean genotypes (Populations based on seed coat colour)

Principal coordinate analyses (PCoA) showed two distinct groups represented by Kala bhat and yellow seed coat colour soybean respectively. The brown seed coat colour soybean got distributed in both the groups. The yellow seed coat colour soybean was confined to one group, a similar pattern was also observed during the cluster analysis. The first three axes of PCoA have explained a cumulative percent variation of 33.15% (Fig. 4). This shows large diversity exists in the genotypes studied.

### Co-linearity between hierarchical cluster and model based population analysis

**Fig. 5:**
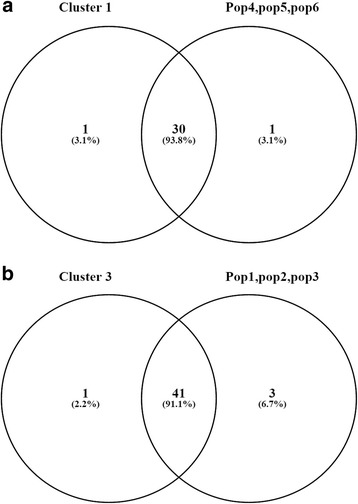
**a** Venn diagram showing co linearity between cluster 1 and pop4, 5, 5 **b** Venn diagram showing co linearity between cluster 3 and pop1, 2, 3

Since the similar pattern of a grouping of genotypes was observed in the hierarchical cluster as well as in population structure, therefore, the Co-linearity between a grouping of genotypes in hierarchical cluster and model based population structure was confirmed using Venn diagram (Fig. 5a and b). The Venn diagram (Fig. 5a) showed that, out of 32 genotypes tested; 30 genotypes were common between population 4, 5, 6 and cluster 1 (93.8%) similarly, Venn diagram (Fig. 5b) showed that 41 genotypes were common between population 1, 2, 3 and cluster 3 (91.1%). This study supports that grouping of soybean genotypes based on the hierarchical cluster and model based approaches were more than 90% similar.

## Discussion

The assessment of genetic diversity is not only important for crop improvement but also important for the efficient management and protection of the available genetic resource. The reliable and authentic results of molecular profiling have made it preferred in genetic diversity study. The molecular study is less influenced by environmental fluctuations, stands another reason for its preference in breeding [28]. Also, it is less biased when compared with estimates obtained by the coefficient of parentage and phenotypic characters [19]. Genetic diversity study has several aspects, first, to identify distinct genetic groups for the retention of germplasm [29], second, to identify genes that correspond to important phenotypic traits and genetic shifts during domestication approach, third, is to find the aspects of history and timing of domestication.

The SSR primers used in the present study amplified an average number of 2.61 alleles per locus with a gene diversity value of 0.43. Li et al. [30] reported 19.7 alleles per locus with gene diversity value of 0.72 during characterization of 1863 Chinese soybean landraces with 59 SSR markers. Similarly, Guan et al. [31] reported 16.2 alleles per locus with a gene diversity of 0.84 while comparing the genetic diversity of 205 Chinese landraces and also Liu et al. [32] reported 7.14 alleles per locus in his study on 91 Shaanxi soybean landraces. These reports show a higher number of alleles per locus in comparison to present study. Doldi et al. [33] reported two to six alleles per locus during characterization of 18 soybean cultivars using 12 microsatellite primers and Tantasawat et al. [34] reported 4.82 alleles per locus. Therefore, allelic richness (average number of alleles per locus) is an effective index for diversity evaluation but it is largely dependent on the sample size [35]. Hence to improve the allelic richness more landraces needs to be introduced into the system thus, enhancing genetic diversity. The mean PIC value obtained in the present study was 0.36, where sat180, sat600, sat554 and sat478 are having 4 alleles per locus and PIC value between 0.55-0.66. These markers with high PIC values become informative for distinguishing among the soybean genotypes. Similar values have been reported by Zhang et al. [36] (0.38), Hisano et al. [37] (0.40), Wang et al. [35] (0.50) and Kim et al. [38] (0.87) with good genetic diversity in their set of samples. As a self -fertilizing crop soybean is expected to have low heterozygosity than hybrid crops [36], here we got low heterozygosity (0.11) much lower than the value reported by Zhang et al. [36] (0.46). Li et al. [30] reported heterozygosity of 0.014 in grain soybean whereas, 0.069 and 0.446 were reported in wild soybean by Liu et al.[39] and Wang et al. [40] respectively. Gene diversity observed in the present study was 0.43; this low level of gene diversity may be ascribed to the emphasis on direct introductions from introduced germplasm and single cross hybrids in the soybean breeding programs. Therefore, diverse germplasm needs to be introduced for more genetic variability [41] Narvel et. al. [14] analyzed 79 elite soybean cultivars with 74 SSR markers showing a low value of gene diversity. Gene diversity reported by Li et al. [42] Wang et al. [43] and Hudcovicova and Kraic [44] showed a substantially higher -value i.e. 0.77, 0.80 and 0.71 respectively on different sets of soybean genotypes. Hierarchical clustering divided the soybean landraces into three distinct clusters, and yellow seed coat colour soybean got grouped into one cluster. In this study, seed coat colour based grouping was more logical than grouping based on geographical location. The analysis based on geographical location showed mixing of genotypes from one location to another location and indicated frequent seed exchange across the geographical location. But when cluster analysis was done based on seed coat colour, the yellow seed coat colour genotypes were grouped together except one genotype(IC-469881). This shows that yellow seed coat colour genotypes are a recent introduction into this area, and breeders have not utilized yellow seed colour genotypes in the breeding programs. Tantasawat et al. [34] reported four major clusters in 25 soybean genotypes analysed by 11 SSR markers. Wang et al. [40] obtained two groups with five wild soybean population assessed by ten SSR markers and Wen et al. [45] also reported two clusters while studying the evolutionary relationship among ecotypes of Glycine *max* and G. *soja* in China. Ghosh et al. [46] reported two clusters and six sub clusters while studying 32 soybean cultivars with 10 SSR markers. Hirota et al. [47] studied black soybean landraces of Tanba region and got two distinct clusters, where as three clusters were obtained by Kondetti et al. [48] while studying 55 Indian Soybean varieties. Population structure divided the soybean genotypes into six different populations. Qiu et al. [49] reported three populations as wild, semi wild and cultivated soybean from Yangstee region whereas; two populations were obtained by Chung et al. [50] in Korean wild and cultivated accessions of soybean and Gyu-Taek Cho et al. [51] reported three populations in Korean land races. PCoA analysis also showed consistent results when seen in terms of a grouping of landraces in cluster analysis. AMOVA showed 12% variance between populations, 22% variance within individuals and 66% variance among individuals. Since soybean is a self pollinated crop, therefore, less variation within individual and more variation among varieties/land races are expected. The analysis done by Venn diagrams showed that, more than 90% co-linearity between cluster 3 and pop1, pop2, pop3 and between cluster 1 and pop4, pop5, pop6. This study proves that SSR based genotyping is a better way to study the genetic diversity in soybean because grouping done by the Hierarchical method and population structure method were more than 90% similar.

## Conclusions

Our study showed that Kala bhat, which has medicinal properties possess large diversity in comparison to yellow and brown seed coat soybean genotypes cultivated in Uttarakhand, India. This study confirms the hypothesis that the landraces are thought to possess rare alleles and therefore, good genetic diversity. This study also provides useful insights about the Kala bhat (black coloured soybean) among different districts of Uttarakhand and simultaneous isolation of yellow coloured soybean. Improving the genetic base requires an introduction of new alleles into the breeding program, and this can only be done by exploiting the genetic variability found in Kala bhat.

## Abbreviations

AMOVA: Analysis of molecular variance
PCoA: Principal Coordinate Analysis
PIC: Polymorphic information content
SSR: Simple sequence repeats
